# Potent and long-lasting humoral and cellular immunity against varicella zoster virus induced by mRNA-LNP vaccine

**DOI:** 10.1038/s41541-024-00865-5

**Published:** 2024-04-04

**Authors:** Anannya Bhattacharya, Lonzaric Jan, Olga Burlak, Jilong Li, Ghanshyam Upadhyay, Katherine Williams, Jinhui Dong, Harrison Rohrer, Michelle Pynn, Andrew Simon, Nathan Kuhlmann, Sergei Pustylnikov, Mariane B. Melo, Antu K. Dey

**Affiliations:** 1 GreenLight Biosciences Inc., 29 Hartwell Avenue, Lexington, MA 02421 USA; 2 Present Address: Icosavax (AstraZeneca), 1930 Boren Avenue, Seattle, WA 98101 USA

**Keywords:** Viral infection, RNA vaccines

## Abstract

Varicella zoster virus (VZV) is a highly contagious human herpes virus responsible for causing chickenpox (varicella) and shingles (herpes zoster). Despite the approval of a highly effective vaccine, Shingrix^®^, the global incidence of herpes zoster is increasing and the economic burden to the health care system and society are substantial due to significant loss of productivity and health complications, particularly among elderly and immunocompromised individuals. This is primarily because access to the vaccines remains mostly limited to countries within developed economies, such as USA and Canada. Therefore, similarly effective vaccines against VZV that are more accessible to the rest-of-the-world are necessary. In this study, we aimed to evaluate immunogenicity and memory response induced by three mRNA-LNP-based vaccine candidates targeting VZV’s surface glycoprotein E (gE). C57BL/6 mice were immunized with each candidate vaccine, and humoral and cellular immune responses were assessed. Our results demonstrate that the mRNA-LNP-based vaccine candidates elicited robust and durable humoral responses specific to the gE antigen. Notably, mice vaccinated with the mRNA-LNP vaccines exhibited significantly higher antigen-specific T-cell cytokine production compared to the group receiving Shingrix^®^, the current standard of care vaccine. Additionally, mRNA-LNP vaccines induced long-lasting memory response, as evidenced by detection of persistent gE-specific Long-Lived Plasma Cells (LLPCs) and memory T cells four months after final immunization. These findings underscore the potential of our mRNA-LNP-based vaccine candidates in generating potent immune responses against VZV, offering promising prospects for their clinical development as an effective prophylactic vaccine against herpes zoster.

## Introduction

Varicella-zoster virus (VZV) is a highly infectious neurotropic human alpha herpesvirus that causes primary varicella infection i.e., chickenpox. In the absence of varicella vaccination, in temperate settings primary infection typically occurs in early childhood^1^, while in tropical settings it often first occurs among adolescents and adults in whom it is associated with significant morbidity and mortality^2^. As the primary varicella infection resolves, the virus remains dormant in the dorsal root ganglia and/or cranial nerves and can reactivate later in life, either due to age-related decrease in immunity or due to immunosuppression, causing herpes zoster (HZ), commonly known as shingles.

During primary infection, viral replication is controlled by innate and adaptive immune responses, including natural killer (NK) cells followed by IgM, IgA, and IgG antibodies and VZV-specific T-cells targeting VZV glycoprotein E (gE), immediate early (IE) 62 protein and other antigens. The primarily T helper type 1 (Th1) cellular response is necessary to disrupt viral replication and enable recovery, and the magnitude and rate of the cellular response inversely correlates with infection severity^3^. Cellular immunity is also critical to limit reactivation and replication of latent VZV. After resolution of primary infection, VZV establishes lifelong latency in sensory, autonomic, and enteric ganglia^4,5^. Multiple cycles of subclinical reactivation and control are posited to occur during a human’s lifetime^6^, and incompletely controlled VZV reactivation leads to herpes zoster. With advancing age and concomitant waning immunity, the incidence, the severity and the extent of debilitating complications due to HZ increases significantly. The most common complication of HZ is postherpetic neuralgia (PHN), precipitated by viral spread from the nerve root throughout the ganglia and characterized by neuropathic pain and dysesthesia that can persist for >1 year in >30% of individuals after HZ resolution and is often refractory to treatment^7–9^. While rarely lethal with a mortality rate of 0.28–0.69 cases per 1 million, HZ causes significant morbidity and societal costs^10^. In the United States alone, it is estimated that HZ results in $2.6 billion in direct medical costs per year^11^. Importantly, given that >95% of the global population older than 50 years of age have prior exposure to VZV, most individuals worldwide are at risk of developing HZ^12^. Without vaccination, individuals who live up to 85 years of age or more have an approximately 50% lifetime risk of developing HZ^13^. Hence, prevention of HZ via vaccination is an important global health priority.

Current treatment options include oral antivirals, such as acyclovir or valaciclovir, which can reduce illness severity and duration but may not reduce rates of PHN or other complications^14,15^. This limitation, as well as the increasing incidence of herpes zoster and its complications, stimulated the development of vaccines to prevent herpes zoster, particularly in older adults and immunosuppressed individuals.

Two vaccines to prevent herpes zoster were approved in the United States: Zostavax® and Shingrix^®^. Zostavax^®^ is a live attenuated vaccine manufactured by Merck Sharp & Dohme, USA, and Shingrix^®^ is an adjuvanted subunit vaccine manufactured by GSK^14,16–19^. The efficacy of Zostavax^®^ is about 64% in 60–69 years old individuals, but the efficacy decreases with increasing age, with only 18% efficacy in individuals greater than 80 years old^14,17–19^. Therefore, Zostavax^®^ has been discontinued in the United States since 2020. Shingrix^®^ contains the recombinant, soluble VZV glycoprotein E (gE), the most abundant glycoprotein on the virion envelope and expressed on the plasma membrane of infected cells, and the adjuvant AS01B. Shingrix^®^ is more than 90% effective at preventing shingles in adults older than 50 years^16,17,20,21^. Despite the high efficacy, the administration immunization of Shingrix^®^ is accompanied by reports of high reactogenicity with frequent solicited reports of injection site pain and severe grade systemic reactions within 7 days after vaccination^21,22^.

We, therefore, aimed to develop a safe and effective mRNA vaccine against herpes zoster that would generate robust virus-specific cell-mediated and humoral immune responses and induce long-term immunological memory. We recently demonstrated the ability of our platform mRNA technology to be safe and induce high immunogenicity against SARS-CoV-2^23^. Here, we designed three versions of the VZV glycoprotein E, gE, encoded by the mRNA: (i) full-length gE (gE-full length), as present on the surface of the virion and naturally infected cells^24^, (ii) truncated gE (gE-truncated), generated by deleting a part of the C-terminal domain and a single amino acid substitution altering one of the trans-Golgi network (TGN) localization motifs, as described previously^25^, and (iii) soluble gE (gE-soluble), which is expressed as the extracellular domain of the protein and similar to the antigen sequence in Shingrix^®^. Upon production of the mRNA-LNP vaccine material and immunization in mice as 2-dose regimen, administered 4 weeks apart, we observed that the GLB mRNA-LNP vaccines encoding the various gE forms are highly effective at inducing both humoral and cell-mediated responses, similar to Shingrix^®^ that was used as a control. We also found that the antigen-specific effector T cell memory responses persisted over 16 weeks after last vaccination, similar to Shingrix^®^. These results, taken together, support the development of the mRNA-LNP formulation delivering the VZV gE full-length antigen as a vaccine candidate against herpes zoster for future clinical evaluation.

## Results

### gE antigen design, expression, and characterization

**Fig. 1:**
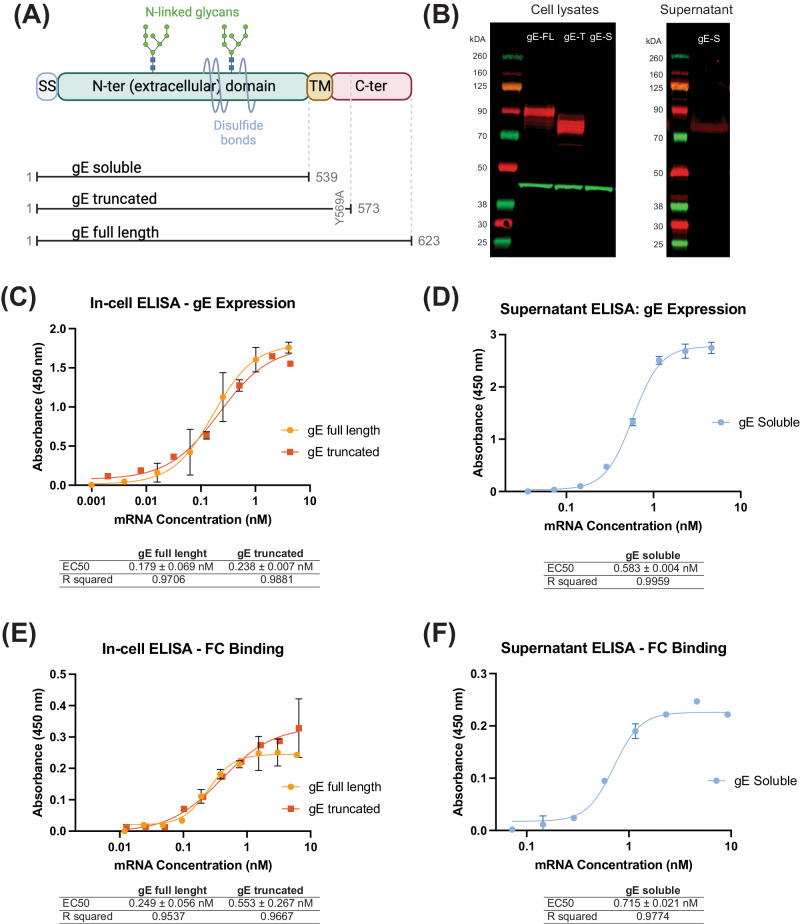
Design and in vitro characterization of mRNA constructs expressing the three different versions of Varicella Zoster Virus (VZV) glycoprotein E (gE). **A** Schematic representation of the various domains of VZV gE. The N-terminal domain contains disulfide bonds, N-glycans, and O-glycans (O-glycans not shown). Three versions of the VZV gE protein that we designed are shown: The soluble gE protein (gE soluble) contains only the signal sequence (SS) and the extracellular N-terminal domain. The truncated gE protein (gE truncated) contains the signal sequence, extracellular N-terminal domain, transmembrane (TM) domain and part of the C-terminal domain with one point mutation in the trans-Golgi network (TGN) localization motif, wherein AYRV sequence was modified to AARV (Y569A). The full-length gE protein (gE full-length) contains the entire wild-type open reading frame of the glycoprotein. **B** Western blot of cell lysates (left) and cell supernatants (right) after transfection with the indicated gE-expressing mRNA constructs. gE-FL – gE full-length, gE-T – gE truncated, and gE-S–gE soluble protein. Red bands correspond to the gE protein, green bands correspond to beta actin that is used as loading control. The observed molecular weight sizes for gE-FL (90 kDa) and gE-T (80 kDa) correspond to their partially glycosylated forms. gE-S (observed at 75 kDa) is not detected in the cell lysates, as expected, but is instead detected in the supernatant (right). **C**, **D** Detection of gE expression on cell surface (gE full-length and gE truncated) and in supernatant (gE soluble) by ELISA. EC50 values (in nM) are shown as Mean ± SD; R square values are shown to highlight the goodness of fit. The detection is performed using anti-gE antibodies. **E**, **F** Binding of human Fc fragment to cell surfaces expressed gE (gE full-length and gE truncated) (**E**) and gE in supernatant (**F**) to demonstrate the appropriate conformation and functional nature of the gE proteins. EC50 values (in nM) are shown as Mean ± SD; R square value is shown to highlight the goodness of fit.

Since glycoprotein E (gE) is the most abundantly expressed antigen both on the surface of the varicella zoster virus (VZV) particles and on the infected cells^24^ and is used in the currently approved subunit vaccine Shingrix^®^^16^, we therefore designed our mRNA constructs to encode this protein. gE is a transmembrane protein consisting of a long N-terminal extracellular domain expressed on the cell or viral surface, a short transmembrane domain anchoring the protein to the viral or host cell membrane, and a short C-terminal cytoplasmic domain. The N-terminal extracellular domain mediates its binding to insulin-degrading enzyme (IDE) on the cell surface^26^ and represents the main VZV antigen for recognition by antibodies and T-cells. The C-terminal domain has been shown to play a critical role in gE protein localization, however, the relationship between localization of the membrane protein and immunity is poorly understood. Therefore, our experimental designs for selection of a particular gE antigen-encoding mRNA candidate were based on comparison of the full-length gE antigen to its truncated variants. Specifically, we designed three versions of the gE antigen (Fig. 1A): (i) full-length gE (gE full-length), as present on the surface of the virion or naturally infected cells^24^, (ii) truncated gE (gE truncated), generated by deleting a part of the C-terminal domain and including a single amino acid substitution (*Y569A*) altering one of the trans-Golgi network (TGN) localization motifs, as described previously^25^, and (iii) soluble gE (gE-soluble), which is expressed as the extracellular domain of the protein and similar to the antigen sequence in Shingrix^®^. In truncated gE, the C-terminal domain containing the trans-Golgi network (TGN) localization motif was modified by a single point-mutation: AYRV to AARV (Y569A). These modifications were made in truncated gE with the aim to address gE trafficking and thus potentially improve the surface expression of gE after translation. In addition to a codon-optimized open reading frame, the mRNA also contained a proprietary transcription initiation sequence (TIS), human β-globin (HBG) 5′ UTR and 3′ UTR sequences, and a polyA tail sequence. The final transcript was 5′ capped and contained N1-methylpseudouridine (m1Ψ) modified bases.

After generation of the mRNA constructs, we successfully demonstrated the expression of all three gE variants in transfected HEK293FT cells by Western blotting (Fig. 1B) and in transfected HeLa cells by in-cell ELISA (for gE full-length and gE truncated; Fig. 1C) and supernatant ELISA (for gE soluble; Fig. 1D). The various gE proteins migrated on the PAGE gel at expected molecular weights with a clear difference between the sizes of the full length and truncated (gE truncated and gE soluble) versions of the protein. As expected, the soluble gE was not detected in cell lysates but was detected in the supernatants of transfected cells both by Western blot (Fig. 1B) and by ELISA (Fig. 1D). In-cell ELISA (gE full-length and gE truncated) and ELISA (gE soluble) showed that the expression levels of full-length gE and truncated gE antigens (Fig. 1C) were similar to the soluble gE antigen (Fig. 1D). This was further confirmed when HEK293FT cells, expressing the three gE antigen variants, were treated with Brefeldin A and we observed similar expression levels via Western blot (data not shown). Additionally, we characterized the functionality of the expressed gE proteins by their ability to bind human Fc. To that end, we developed Fc-binding ELISAs and observed that all three gE variants bound the recombinant human Fc similarly (Fig. 1E for gE full-length and gE truncated antigens binding to Fc; Fig. 1F for gE soluble antigen binding to Fc). These results confirmed the successful designs of the mRNA constructs encoding the three gE antigen variants, their appropriate expression upon transfection, and demonstration of functional antigenic conformation via human Fc-binding.

### Production of mRNAs and mRNA-LNP vaccine candidates for in vivo studies

To generate vaccine candidate materials for the in vivo studies, the three gE-encoding (gE full length, gE truncated and gE soluble protein) mRNAs were produced by in vitro transcription, purified using lithium chloride precipitation, and stored at ≤−65 °C until further use. The purified mRNAs were tested for the following key attributes: concentration, identity, purity/integrity, poly(A) tail length, % capping efficiency, % N1-methyl-pseudouridine (m1Ψ) incorporation, and levels of various impurities (e.g., RNase, endotoxin, *E.coli* DNA and residual NTPs). These analytical test results are shown in Supplementary Table S2. Thereafter, all three purified gE-encoding mRNAs were encapsulated in either NOF-lipid-based LNPs or SM102-lipid-based LNPs and the final formulated mRNA-LNPs were tested for the following critical attributes: Particle size, Polydispersity index (PDI), % mRNA encapsulation, % mRNA purity post encapsulation and mRNA concentration. The analytical test results of the six mRNA-LNPs are shown in Supplementary Table S3.

### Assessment of in vivo delivery and preliminary immunogenicity of GLB mRNA-LNP vaccine candidates

We aimed to evaluate immunogenicity of the three gE-encoding GLB mRNA-LNP vaccine candidates in mice. However, before evaluating all three gE variants, we wanted to conduct a preliminary study using the mRNA-LNP formulation encoding the full-length glycoprotein E (gE). The LNP formulation was based on proprietary cationic lipids from NOF corporation. The aim of this preliminary study was to quickly test if the novel NOF-LNP formulation was effective in delivering the mRNA in vivo, using the gE full-length antigen, before conducting the full comparative evaluation of the gE variants. As positive control, the currently approved subunit vaccine Shingrix^®^ was used.

**Fig. 2:**
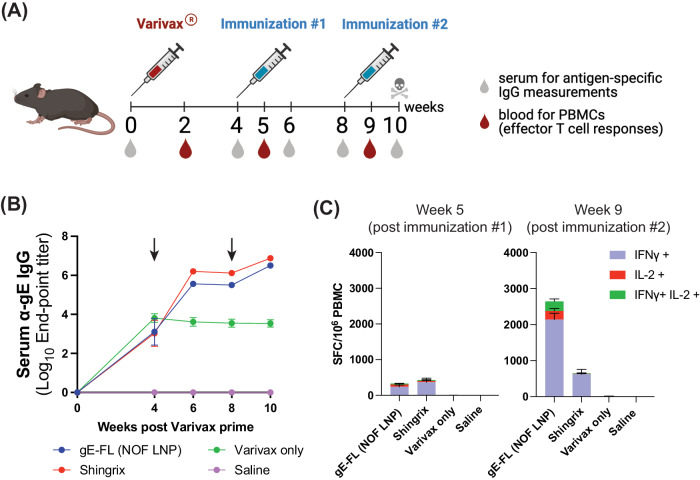
Initial immunogenicity of mRNA-LNP vaccine encoding for the VZV full-length gE (gE full-length) in mice. Female C57BL/6 mice were primed with a full human dose of the live attenuated VZV vaccine (Varivax^®^) by subcutaneous administration. Mice were then injected intramuscularly with 5 μg of mRNA-LNP vaccine candidates, expressing VZV gE full-length, or saline (Varivax^®^ only group) at weeks 4- and 8- post Varivax^®^ administration. NOF-LNP formulated mRNA, delivering gE full-length, is the test candidate while Shingrix^®^ is used as a positive control. **A** Scheme of immunizations and sample collections schedule. **B** End-point titers of gE-specific IgG binding antibodies detected in mice at the indicated timepoints and evaluated by ELISA. Arrows indicate the two mRNA-LNP/Shingrix^®^ immunization timepoints. Data shown as Mean ± SEM, and is representative of two independent experiments using 10 animals per group. Y-axis represents Log_10_ end-point titers; *X*-axis represents weeks post-Varivax^®^ prime. **C** Antigen-specific effector T cell responses measured from whole blood using a murine IFN-γ/IL-2 Double-Color Enzymatic ELISpot Assay. Data shown as Mean ± SEM. Y-axis represents Spot Forming Cells (SFCs) per million peripheral blood cells. Each SFC corresponds to an effector T cell secreting either one or both cytokines in response to stimulation by the gE overlapping peptides pool.

To mimic pre-existing immunity against VZV, which is expected to be present in dormant state in the target human population, C57BL/6 female mice were primed with a full human dose (500 μL) of the live-attenuated Varicella Zoster virus (Varivax^®^) by subcutaneous administration. It should be noted that the live-attenuated VZV (Varivax^®^) cannot replicate in mouse cells and establish latency in this animal. Mice were immunized with two intramuscular injections of the gE full-length encoding mRNA-LNP (NOF) candidate, 4- and 8-weeks post Varivax^®^ priming, as shown in Fig. 2A. Immunizations were performed at a dose of 5 μg per animal, in a total volume of 50 µL per mouse (25 µL in each hind limb). Mice injected with Varivax^®^ only or saline were included as controls.

Anti-gE IgG binding antibodies were detected by ELISA from serum samples collected at 0-, 4-, 6-, 8-, and 10-weeks post Varivax^®^ prime. Immunization of mice with 5 μg of gE full-length mRNA-LNP (NOF) induced high levels of anti-gE serum IgG binding antibodies that were comparable to those elicited by Shingrix^®^, as shown in Fig. 2B. Anti-gE binding antibody titers observed in animals immunized with mRNA-LNP (NOF) vaccine increased significantly after each immunization, when compared to animals primed with Varivax^®^ only.

Effector T cell responses were assessed from peripheral blood 2 weeks post Varivax^®^ prime (baseline response), and 1 week post the first and second mRNA-LNP immunizations using the murine IFN-γ/IL-2 Double-Color Enzymatic ELISpot Assay. gE-specific activated T cells secreting either IFN-γ or IL-2 or both cytokines, after re-stimulation with gE overlapping peptides, were quantified (Fig. 2C). At baseline, the number of spot-forming cells (SFC) in all groups were similarly low, below 10 SFC per million blood cells. However, following two immunizations with 5 μg of mRNA-LNP (NOF) vaccine candidate, all animals developed strong gE-specific effector T cell responses that were higher than those observed in mice administered with two injections of Shingrix^®^. No long-lasting T-cell response was detected in animals primed with Varivax^®^ alone. Taken together, the data from this preliminary study shows that the GLB mRNA-LNP (NOF) formulation is successful in delivering the gE glycoprotein and successful in eliciting a strong immunogenic response, comparable to Shingrix^®^, in mice.

### Assessment of comparative immunogenicity of GLB mRNA-LNP vaccine candidates encoding the three different versions of gE antigen

Based on the success of the above preliminary in vivo study in mice, we designed this expanded comparative immunogenicity study to evaluate all three mRNA-LNP vaccines candidates, encoding the three different versions of the gE antigen, and to compare them to Shingrix^®^. For this study, the three gE antigen variants-encoding mRNAs were formulated in two different LNP formulations for in vivo testing: one using the proprietary NOF LNP and the other using SM102 LNP, as a LNP comparator. SM102 LNP was chosen as a comparator because successful delivery of mRNA encoding for VZV’s gE by SM102 LNP has been reported earlier by Monslow et al.^27^. Each mRNA-LNP candidate was produced, tested for critical attributes (mRNA content, mRNA integrity and purity, LNP size and polydispersity) and thereafter frozen at <−65 °C until use in the study. In this study, two different antigen doses for each of the gE antigens were tested: a high dose of 5 µg and a low dose of 1 µg.

**Fig. 3:**
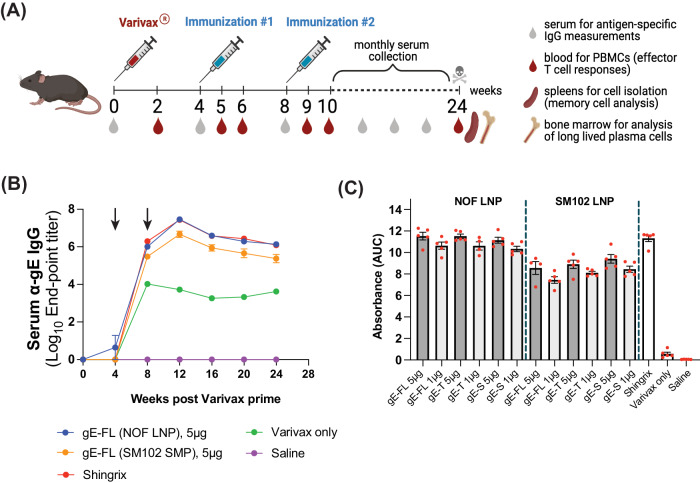
Comparative immunogenicity of mRNA-LNP vaccine candidates encoding three different versions of VZV gE antigen. Female C57BL/6 mice (5 animals per group) were primed with a full human dose of the live-attenuated VZV) vaccine (Varivax^®^) by subcutaneous administration. Mice were then injected intramuscularly with 1 or 5 μg of mRNA-LNP vaccine candidates, encoding the three gE variants, or saline (Varivax^®^ only group) at 4 and 8 weeks post Varivax^®^ administration. **A** Scheme of immunization and sample collection schedule. **B** Longitudinal Log_10_ end-point titers (Y-axis) of gE-specific IgG binding antibodies detected in sera collected from mice at the indicated timepoints and evaluated by ELISA. Arrows indicate mRNA-LNP/Shingrix^®^ immunization time points. Data shown as Mean ± SEM. **C** Quantitative assessment of anti-gE serum IgG binding antibodies in mice from all vaccinated groups at 12 weeks post Varivax^®^ prime, assessed by ELISA and expressed as area under the curve (AUC) (Y-axis) based on absorbance values. Data shown as Mean ± SEM.

Consistent with the previous mouse study (Fig. 2A), this study in C57BL/6 female mice was also similarly designed. All mice received a full human dose (500 μL) of the live attenuated Varicella vaccine (Varivax^®^) by subcutaneous administration to set the initial VZV infection. 4 weeks post Varivax^®^ priming, mice were administered with two intramuscular injections of the formulated mRNA, 4 weeks apart (Fig. 3A). Additionally, one group of mice was administered with 5 µg of Shingrix^®^ (1/10 of the human dose) twice, 4 weeks apart, as positive control or an active comparator. Vaccines were administered in a total volume of 50 µL per mouse (25 µL in each hind limb). Mice injected with Varivax^®^ only and saline were included as (negative) controls.

Anti-gE binding antibodies were detected by ELISA from sera of mice collected on 0, 4, 8, 12, 16, 20, and 24 weeks. Serum IgG binding antibody levels induced by NOF LNPs encapsulating 5 µg of mRNA were equivalent to antibody levels observed in animals vaccinated with Shingrix^®^ (Fig. 3B). Moreover, NOF LNP-formulated vaccine induced higher levels of gE-specific binding antibodies when compared to SM102 LNP-formulated vaccines in a dose-dependent manner (Fig. 3C). The peak binding antibody response was observed at week 12 i.e., 4 weeks post 2nd vaccination (Fig. 3B, C). As expected, mice vaccinated with saline had no detectable anti-gE serum IgG throughout the study.

**Fig. 4:**
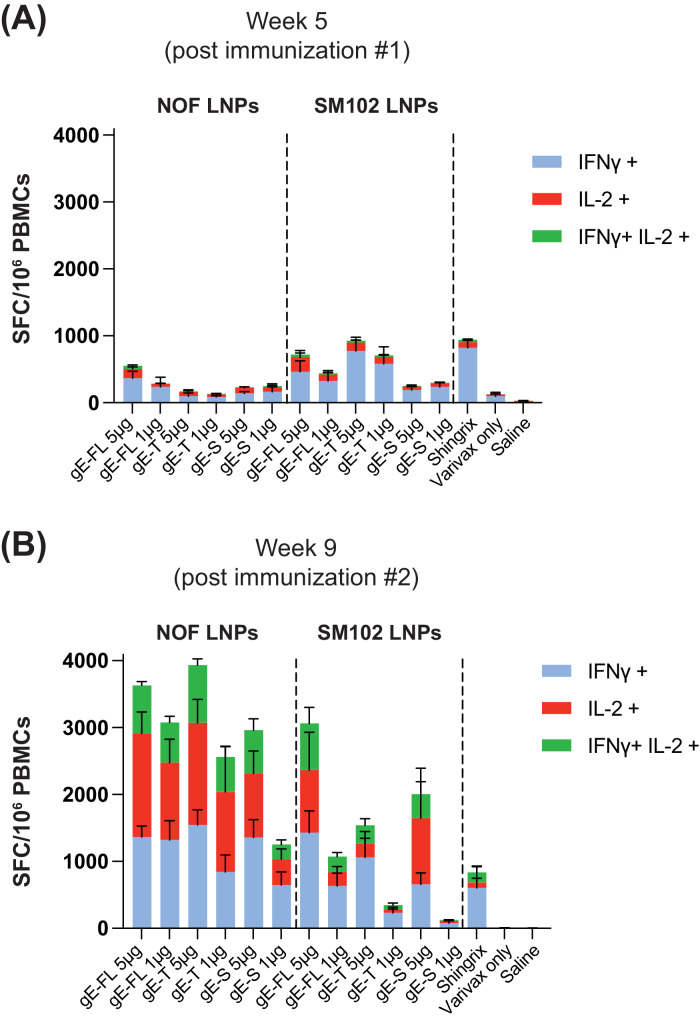
mRNA-LNP vaccine candidates, encoding VZV gE, induces strong antigen-specific T cell responses. **A**, **B** Female C57BL/6 mice (5 animals per group) were primed with a full human dose of the live attenuated VZV vaccine (Varivax^®^) by subcutaneous administration. Mice were injected intramuscularly 5 μg of the mRNA-LNP vaccine candidates, expressing the three gE variants, or Shingrix^®^ at 4 and 8 weeks post Varivax^®^ administration. **A**, **B** Antigen-specific effector T cell responses were measured from whole blood cells collected at week 5 i.e., 1 week post 1st immunization (**A**) or week 9, i.e., 1 week post 2nd immunization (Busing a murine IFN-γ/IL-2 Double-Color Enzymatic ELISpot Assay. Data shown as Mean ± SEM. Y-axis in both panels, **A** and **B**, represents Spot Forming Cells (SFCs) per million peripheral blood cells. Each SFC corresponds to an effector T cell secreting either one or both cytokines in response to the gE overlapping peptides pool.

Previous studies suggest that CD4^+^ T cells play a critical role in protecting against reactivation of VZV^28,29^. The decline in VZV-specific CD4^+^ T-cell responses has been associated with an increased risk of herpes zoster in older adults^30,31^. Moreover, immunocompromised individuals with impaired CD4^+^ T-cell function, such as people living with HIV, but not antibody deficiencies, are at a higher risk of developing herpes zoster and are more likely to experience severe and prolonged episodes^28^. Therefore, vaccines that can boost CD4^+^ T cell responses are believed to provide additional protection against reactivation of VZV. To that end, to assess amplitude and kinetics of T cell responses induced by the various mRNA-LNP vaccines encoding the three gE antigen variants, peripheral blood was collected 2 weeks post Varivax^®^ prime, and at 1 and 2 weeks post each vaccination (mRNA-LNP or Shingrix^®^), as depicted in Fig. 3A. Peripheral blood mononuclear cells were stimulated with a gE overlapping peptide pool, and the number of gE-specific cells secreting IFN-γ and/or IL-2 was quantified by dual-color ELISpot. Priming with Varivax^®^ induced very weak T-cell responses in mice (Fig. 4A), as also previously described by others^19,27^. Effector T cell responses peaked at day 7 post each vaccination, either by the mRNA-LNP vaccines or the active control (Shingrix^®^). Overall, the mRNA-LNP vaccine expressing the soluble version of gE performed poorly when compared to mRNA-LNP vaccines encoding the full-length gE or the truncated gE; regardless of the type of LNP (NOF vs. SM102) formulation used (Fig. 4A, B). Remarkably, the three NOF LNP-formulated vaccines encoding each of the three gE antigen variants, induced very high levels of poly-functional effector T cells, evidenced by secretion of more than one cytokine simultaneously, which were higher than levels induced by the Shingrix^®^ vaccinated animals.

**Fig. 5:**
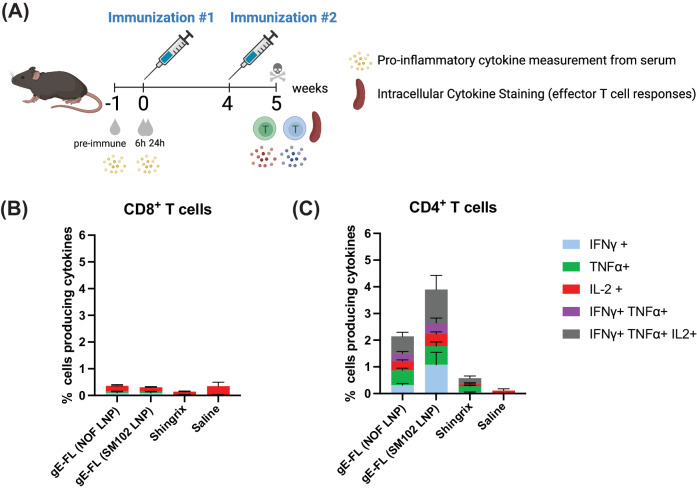
CD4^+^ T cell-biased cellular response induced by gE mRNA-LNP vaccines. **A** Animals were divided in groups of 7, and subsequently immunized with 2 intramuscular doses, 4 weeks apart, of gE mRNA-LNP (NOF or SM102) or Shingrix^®^. Mice injected with saline were included as negative controls. One week after the last immunization, spleens were harvested and stimulated with overlapping peptide pools from VZV gE protein, and percentage of CD8^+^ (**B**) and CD4^+^ (**C**) T cells producing IFN-γ, TNF-α, and IL-2, was measured by flow cytometry.

To bolster the above findings, we conducted an additional study to measure effector T cell responses from splenocytes, collected 1 week post 2nd immunization with mRNA-LNP (NOF), mRNA-LNP (SM102) or Shingrix^®^, by intracellular cytokine staining (ICS). In this study, mice were administered with two intramuscular injections of the LNP (NOF or SM102) formulated mRNA or Shingrix^®^, 4 weeks apart, without Varivax^®^ priming (Fig. 5A). Mice injected with saline were included as (negative) controls. Splenocytes obtained a week after the second immunization were stimulated with gE overlapping peptide pool, and the number of gE-specific CD4^+^ or CD8^+^ T cells secreting IFN-γ, TNF-α and/or IL-2 were quantified by flow cytometry (Supplementary Figure S1). The gE-specific effector T cell responses were predominantly CD4^+^ T cell-mediated, since less than 0.5% of the CD8^+^ T cells in the spleen detected were secreting cytokines upon stimulation with gE peptides (Fig. 5B and Supplementary Figure S2A). Administration of NOF or SM102 formulated mRNA-LNP vaccine candidates induced potent CD4^+^ T cell responses, as seen by the number of gE-specific CD4^+^ T cells secreting either one, two or all three of the cytokines IFN-γ, TNF-α and IL-2 (Fig. 5C and Supplementary Figure S2B). Importantly, both NOF and SM102-formulated mRNA-LNP vaccines induced higher levels of CD4^+^ T cell responses than Shingrix^®^.

### Evaluation of immunological memory induced by GLB mRNA-LNP vaccine candidates

**Fig. 6:**
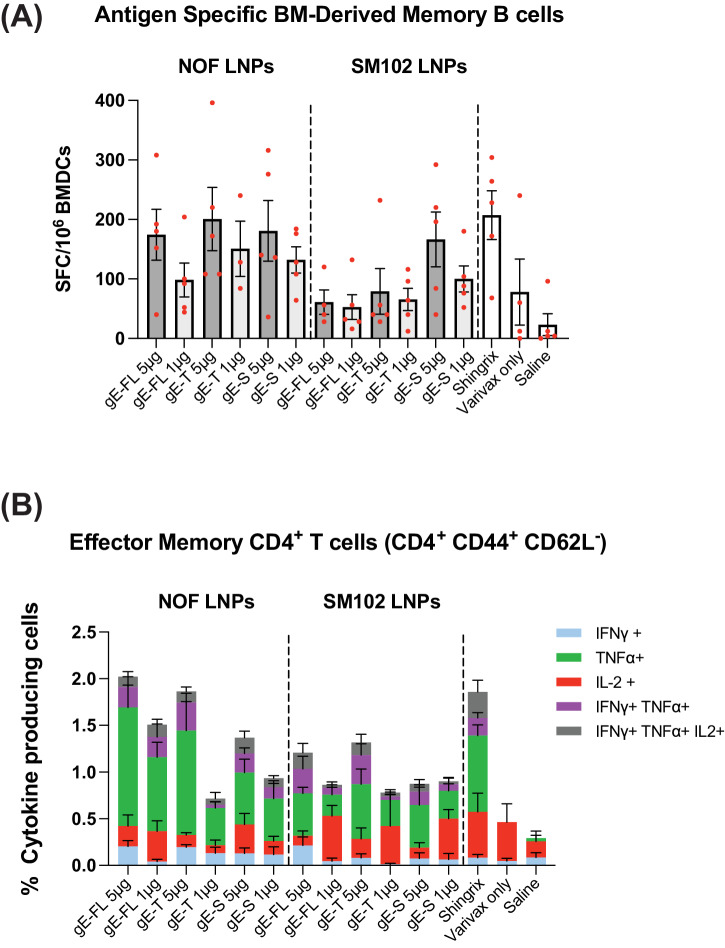
mRNA-LNP vaccine candidates, encoding VZV gE, induces antigen-specific LLPCs and T (T_MEM_) cell memory responses in mice. C57Bl/6 mice (5 animals per group) were primed with live attenuated VZV vaccine (Varivax^®^), and vaccinated twice, 4 weeks apart, with the mRNA-LNP vaccine candidates or Shingrix^®^. Immunological memory was evaluated 16 weeks post last immunization. **A** Long lived plasma cells in the bone marrow secreting antibodies that bind to VZV gE, quantified by B cell ELISpot assay. Y-axis represents Spot Forming Cells (SFCs) per million bone marrow-derived cells. **B** Percentages of effector memory CD4+CD44+CD62L- T cells (Y-axis) secreting either IFN-γ, TNF-α, and IL-2 alone or any of the two or all three cytokines. Data shown as Mean ± SEM.

Effective vaccines are expected to generate adaptive immunological memory in the B cell (B_MEM_) and T cell (T_MEM_) memory compartments. This memory is crucial for rapid recall of the immune responses to avoid re-activation of VZV and causation of herpes Zoster disease^27,32^. To assess if vaccination with the gE antigen encoding mRNA-LNP vaccine candidates could lead to long-term persistence of humoral responses, the presence of gE-specific long-lived plasma cells was investigated in the bone marrows and effector memory CD4^+^ T cells in the spleens of mice, collected 16 weeks post 2nd immunization, as depicted in schematics on Fig. 3A. gE-specific long-lived plasma cells (LLPCs), quantified as the number of bone marrow-derived anti-gE antibody (IgG) secreting cells (ASC) by B cell ELISpot assay, induced by 5 µg of mRNA formulated with NOF LNP, were comparable to responses observed in animals vaccinated with Shingrix^®^ (Fig. 6A). Additionally, gE-specific CD4^+^ effector memory T cells, characterized as CD4^+^ CD44^+^ CD62L^-^, were quantified from spleens. We observed that gE-specific effector memory CD4^+^ T cell responses, induced by 5 µg of mRNA formulated with NOF LNP, were comparable to responses observed in animals vaccinated with Shingrix^®^ (Fig. 6B). Cumulatively, based on the overall gE-specific antibody titers, antigen-specific T cell responses and LLPCs and T cell memory results, we selected the gE full-length for future studies to support clinical development of this vaccine candidate.

### Assessment of pro-inflammatory cytokines induced by GLB mRNA-LNP Shingles vaccine candidates

Despite the high efficacy of Shingrix^®^, vaccinees have reported severe local and systemic reactions post vaccination^21,22^. Thus, aware of the pronounced reactogenicity of Shingrix^®^, one of our major goals was to develop a mRNA-LNP vaccine against herpes zoster that is at least comparable with respect to immunogenicity of Shingrix^®^ but with potentially lower induction of systemic inflammation.

**Fig. 7:**
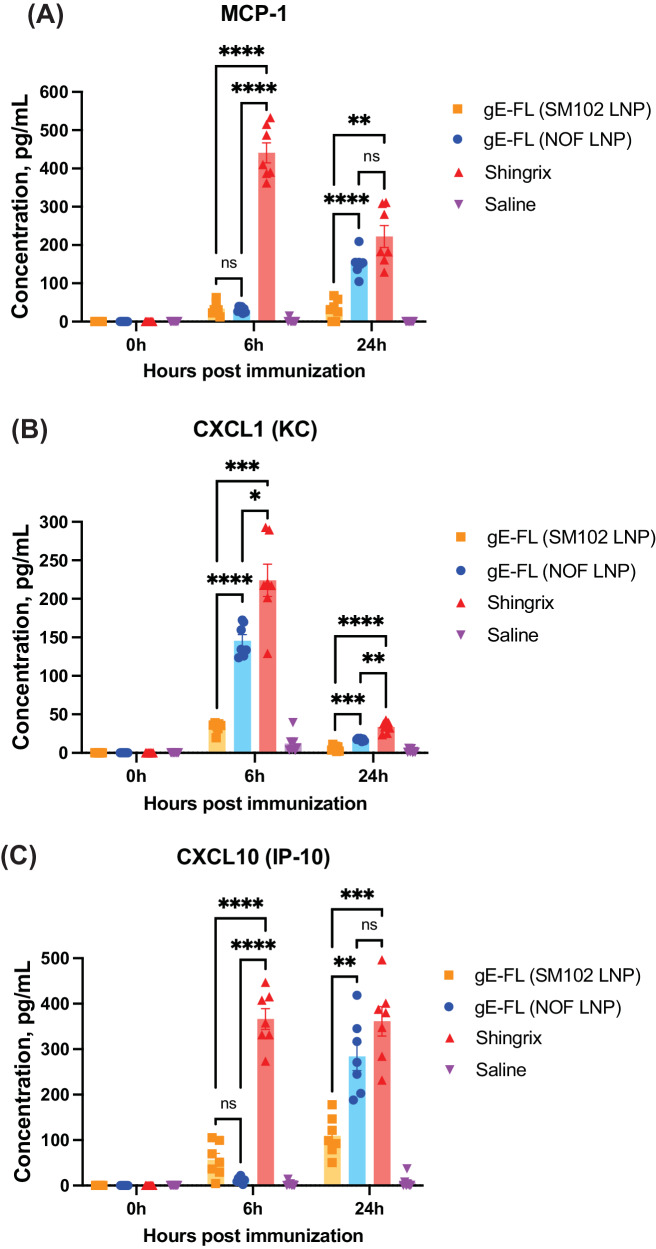
mRNA-LNP vaccine candidates, encoding VZV gE, induce lower levels of inflammatory cytokines in mice than Shingrix^®^. Female C57BL/6 mice (7 animals per group) were immunized with 5 µg of either SM102 LNP-formulated mRNA encoding gE full-length or NOF LNP-formulated mRNA encoding gE full-length or Shingrix^®^ or saline alone (negative control). Pro-inflammatory cytokines (MCP-1 in panel **A**, CXCL1/KC in panel **B**, and CXCL10/IP-10 in panel **C**) were quantified in serum of immunized animals pre-dose, 6 h and 24 h post dosing, using LEGENDplex™ Mouse Anti-Virus Response flow-based multiplexed assay. Y-axis denotes the cytokine concentrations in pg/mL. Data shown as Mean ± SEM. Two-way Analysis of Variance (ANOVA) with Tukey’s multiple comparison test was performed to determine statistical significance. *p < 0.05, **p < 0.005, ***p < 0.0005, ****p < 0.0001; ns - not significant. MCP-1 monocyte chemoattractant protein-1, CXCL1/KC chemokine (C-X-C motif) ligand 1/keratinocyte-derived chemokine, CXCL10/IP-10 chemokine (C-X-C motif) ligand 10/ IFN-γ-inducible protein 10.

To assess systemic inflammation induced by the selected gE full-length mRNA-LNP vaccine candidates, pro-inflammatory cytokines were measured from sera collected before immunization and 6 h and 24 h after the 1^st^ immunization with mRNA-LNP (NOF) formulated vaccine candidate or mRNA-LNP (SM102) formulated vaccine candidate or Shingrix^®^, using the LEGENDplex™ Mouse Anti-Virus Response Panel. Interestingly, both NOF and SM102 lipid-formulated mRNA-LNP vaccines induced significatively lower levels of pro-inflammatory cytokines such as MCP-1 (Fig. 7A), CXCL-1 (Fig. 7B), and CXCL-10 (Fig. 7C) post vaccination than Shingrix^®^. Other pro-inflammatory cytokines such as interferons, IL-1β and IL-6 (that were included in the LEGENDplex™ Mouse Anti-Virus Response Panel) were not detected at any of the time points across all groups. These findings suggest that additional investigations will be necessary in future to allow for the full assessment of local and systemic toxicity of our mRNA-LNP in comparison to Shingrix^®^.

## Discussion

In a previous study^23^, we demonstrated the validity of GreenLight Biosciences’ mRNA platform technology by inducing potent and long-lasting humoral and cell-mediated immunity against SARS-CoV-2 and confirming the safety of the mRNA-LNP formulation. Here, we used the mRNA-LNP technology for screening of top VZV gE antigen variants and evaluating antigen-specific immune responses in mice to allow selection of the top mRNA-LNP vaccine candidate for future clinical development.

Assessment of comparative immunogenicity of the NOF LNP-formulated mRNA vaccine candidates, encoding the three different versions of gE antigens, showed that all three gE-antigens elicited equivalent levels of gE-specific binding antibodies that were comparable to those elicited by Shingrix^®^. Importantly, in our study, we observed that truncation and mutagenesis of the C-terminal domain of gE in our truncated gE antigen, intended to modulate protein localization in the trans-Golgi network, did not improve its immunogenicity, particularly when compared to the full-length gE antigen. In addition, the three NOF LNP-formulated mRNA vaccines also induced very high levels of poly-functional effector T cells, which were higher than levels induced by Shingrix^®^. To evaluate immunological memory, we measured both levels of antibody-secreting cells (ASCs) in bone marrow, and presence of memory T cells in spleens 16 weeks post immunization. ASCs encompasses both short-lived and long-lived plasma cells^33–35^. These long-lived plasma cells (LLPCs), primarily residing in bone marrow, originate within germinal centers following vaccination and typically display high affinity for its cognate antigen^36–38^. These cells meet the criteria of memory cells as they sustain continuous antibody secretion autonomously, irrespective of their precursor cells (B cells), T cell assistance, or antigen presence^33,39^. Thus, LLPCs play a crucial role in preventing reinfection and safeguarding barrier sites from external threats. We found that gE-specific LLPCs responses induced by the three NOF LNP-formulated mRNA vaccine candidates were comparable to responses observed in animals vaccinated with Shingrix^®^. Likewise, the quantities of CD4+ effector memory T cells four months after vaccination with NOF LNP-formulated mRNA vaccine candidates were equivalent to the responses seen in animals inoculated with Shingrix^®^. Taken together, these results confirm that our NOF LNP-formulated mRNA vaccine candidates were successful in generating potent and long-lasting humoral and cellular immune responses against VZV gE, comparable to Shingrix^®^.

In addition to demonstration of strong immunogenicity, we also wanted to assess the systemic pro-inflammatory responses induced by our mRNA-LNP vaccine candidate. This was particularly important because despite the high efficacy of Shingrix^®^, 1 out of 10 individuals experience a grade 3 severe reaction owing to the presence of AS01B adjuvant in the vaccine^21,22^. Reactogenicity embodies the physical display of the inflammatory reaction triggered by vaccination. Vaccines’ induction of innate responses generates inflammatory mediators circulating in the body, including chemokines and cytokines, which are potential causative agents of the signs and symptoms of inflammation at the injection site in the vaccinated individual, such as pain, redness, and swelling^40^. Evaluation of our NOF LNP-formulated mRNA vaccines showed that they induced lower levels of pro-inflammatory cytokines when compared to Shingrix^®^. Nevertheless, further research aimed at assessing the local and systemic toxicity of our mRNA-LNP vaccines in suitable animal models will be necessary to provide insights into their safety profile, facilitating the continued clinical development of these vaccines.

Previous studies have indicated that T-cell-mediated immunity, particularly antigen specific CD4^+^ T cells, are critical in protecting the host from reactivation of latent VZV^41,42^. Interestingly, we observed strong induction of IL-2 in addition to IFN-γ upon administration of two doses of our mRNA vaccine candidates. This is corroborated by the frequency of gE-specific effector CD4^+^ T cells detected in the spleens of mice administered with two doses of the mRNA vaccine candidates, implying that our mRNA vaccine candidates generated a potent CD4^+^ T cell response in an antigen specific manner. Importantly, gE-specific CD8^+^ T cell responses were hardly detected, as previously reported^19^. In line with these findings, we observed comparable effector memory T cell responses between mice administered with 5 µg of NOF LNP-formulated mRNA or Shingrix^®^. Notably, the frequency of CD4^+^ effector memory T cells was higher than that of CD8^+^ effector memory T cells (data not shown), indicating that the memory response is predominated by CD4^+^ T cells rather than CD8^+^ T cells. This is in agreement with a previous study that evaluated cell mediated immune responses in mice after administration of vaccine candidates containing a recombinant gE antigen using the live attenuated VZV-primed mouse model and reported undetectable levels of CD8^+^ T cells in the spleens of vaccinated mice^27^. Consistent with these observations, additional studies have shown that the memory T cell response against VZV in immune adults is characterized primarily by antigen-specific CD4^+^ T cells possessing a Th1-like phenotype, which secrete IFN-γ and IL-2, and very low frequencies of CD8^+^ T cells^41,43–47^.

While the focus on T cell immunity in combatting VZV reactivation is essential, it is also important to assess the protective potential of anti-VZV antibodies produced through vaccination. Early research has established a link between increased anti-VZV antibody levels and a decreased risk of herpes zoster^48^. Here, we observed that NOF LNP-formulated mRNA vaccine induced similar levels of gE-specific antibodies compared to Shingrix^®^, but notably higher antibody responses than induced by the SM102 LNP-formulated mRNA vaccine. Previous studies have shown that vaccination with Zostavax^®^ elicited antibodies targeting various VZV proteins and that anti-gH antibodies demonstrated superior neutralization capabilities, inhibiting cell-to-cell spread^49^. On the other hand, vaccination with Shingrix^®^ specifically elicited high-avidity anti-gE antibodies^50^ and proved more effective in preventing VZV reactivation^21^. Therefore, we believe that further evaluating the functionality of the anti-gE antibodies produced by our mRNA-LNP vaccine candidates can significantly enhance our understanding of gE-antibody-mediated protection mechanisms. Additionally, ionizable lipids within lipid nanoparticles inherently possess adjuvant properties^51^, and different lipids have the potential to activate distinct innate immune pathways, enhancing their adjuvanticity^52^. In this study, a proprietary ionizable lipid from NOF Corporation was employed. Although the mechanism of action of this particular novel LNP has not been described, we can hypothesize that NOF-based LNPs can potentiate antibody generation more efficiently than SM102-based LNPs. Additionally, the kinetics of antigen exposure can significantly affect germinal center development and the quality of antibodies generated^53^. We cannot exclude the hypothesis that different LNPs will release mRNA at different rates, resulting in different kinetics of antigen expression and presentation. Future comparison of germinal center responses, induced by different LNP-based mRNA vaccines, can therefore help elucidate mechanism of antibody production and protection.

In summary, we designed and evaluated the immunogenicity of three different mRNA-LNP vaccine candidates that encoded for three distinct VZV gE antigen variants. All three candidates, when delivered via novel NOF LNPs, were well-tolerated in rats and highly immunogenic and induced potent humoral, cellular, and immunological memory responses in mice. Importantly, we also showed that the NOF LNPs, delivering the gE full-length mRNA, was less inflammatory than Shingrix^®^ and therefore could demonstrate better safety profile in human clinical studies. Based on the overall immunogenicity results in mice and initial safety and tolerability observations in rats for the administered doses of mRNA and NOF LNP formulations, we selected the gE full-length antigen and NOF LNP formulation for next stages of clinical development. While LNPs, using SM102 cationic lipid, for mRNA vaccine delivery and development against multiple infectious disease targets, including VZV^27^, has been evaluated in pre-clinical and clinical stages, this is the first report evaluating the pre-clinical safety and immunogenicity potential of the novel NOF-lipid based LNPs for mRNA vaccine delivery and development, particularly against an infectious disease target (here VZV). Thus, this study provides the mRNA vaccine field with a novel LNP formulation for delivery of mRNAs for future prophylactic and therapeutic applications. Finally, mRNA-based vaccine development against herpes zoster is necessary and an important strategy towards solving global supply of an effective vaccine against the disease that today is hindered by production issues of Shingrix^®^, contributed largely by limited supply of the naturally sourced saponin component QS-21^54^ in the AS01B adjuvant. In addition to addressing the critical issue of global supply of Shingrix^®^, an effective and better-tolerated mRNA vaccine against herpes zoster can be a strong alternative to mitigate the increased reactogenicity associated with administration of Shingrix^®^, particularly the second dose, to the elderly^48^.

## Methods

### Cloning and mRNA preparation

DNA fragments encompassing the coding sequence (CDS) and the 5’ and 3’ untranslated regions (UTRs) were synthetized by Integrated DNA Technologies (IDT), PCR amplified, and cloned into a proprietary linearized vector by Gibson Assembly. The vector backbone contained an ampicillin resistance gene, a high copy origin of replication and a T7 promotor with an upstream terminator for transcriptional insulation. The polyA tail sequence was added by a restriction digestion and ligation reaction. The sequence of the glycoprotein E (gE) was derived from the Oka strain of Varicella Zoster Virus (VZV) and codon optimized. The plasmid DNAs were sequence verified, linearized by digestion immediately downstream of polyA, and transcribed in vitro using a proprietary method and internally produced and purified T7 polymerase. The reaction mixtures contained CleanCap^®^ Reagent AG and N1-methyl-pseudouridine-5’-triphosphate (m1Ψ) instead of Uridine-5’-Triphosphate (UTP). The mRNA was purified using Lithium Chloride (LiCl) precipitation and stored at ≤-65°C until encapsulation in lipid nanoparticles (LNP).

### Cell lines

HEK293FT (ThermoFisher) and HeLa (ATCC) cells were cultured in Dulbecco’s modified Eagle’s medium (DMEM, Gibco) supplemented with 10% fetal bovine serum (FBS, Gibco), 1% penicillin–streptomycin (Gibco), 6 mM L-glutamine (Gibco), 1 mM MEM Sodium Pyruvate (Gibco), 0.1 mM MEM Non-Essential Amino Acids (Gibco) and by incubating at 37 °C with 5% CO_2_. For specific experiments, the cells were transfected with quantified amounts of the mRNA candidates using Lipofectamine™ MessengerMAX™ and cultured for 24 h before harvesting the cells/supernatant and analyzing for gE expression.

### Western blot

24 h after transfection, cells were washed with phosphate-buffered saline (PBS) and lysed with RIPA (Radioimmunoprecipitation assay) buffer with protease inhibitors (Thermo Fisher) and DNAse I (NEB). The lysates or cell supernatants were mixed with western blot (WB) loading dye (NuPAGE™ LDS Sample Buffer) and reducing agent (NuPAGE™ Sample Reducing Agent), denatured by heating, and loaded onto polyacrylamide gels (Bolt™ Bis-Tris Plus Mini Protein Gels, 4‐12%). The proteins were transferred to a PVDF membrane using the BioRad Trans-Blot Turbo system. The membranes were blocked with Intercept Tris-buffered saline (TBS) Blocking Buffer (LI-COR), stained with anti-gE primary antibody (GeneTex), and a goat anti-mouse fluorescently labeled secondary antibody (Invitrogen). The membranes were imaged on a LI-COR Odyssey.

### In-cell ELISA and Fc-binding assays

HeLa cells were transfected with varying concentrations of mRNA, expressing either full-length gE or truncated gE protein using Lipofectamine™ MessengerMAX™ according to manufacturer’s instructions. 24 h after transfection, cells were fixed using a 4% paraformaldehyde (PFA) solution. For in-cell ELISA, gE expression was probed using a gE-specific monoclonal antibody (GeneTex, 1:1000 dilution) followed by detection with secondary antibody conjugated to Horseradish peroxidase (HRP, Abcam, 1:1000 dilution). Alternatively, to detect Fc-binding to the gE proteins, fixed cells were incubated with 4 μg/ml of biotinylated human IgG1 Fc protein (Abcam) for 1 h, followed by detection with streptavidin conjugated to Horseradish Peroxidase (HRP, 1:1000 dilution; Abcam). Tetramethylbenzidine (TMB) was used as a substrate and reaction was stopped with 1 M Phosphoric acid (H_3_PO_4_). Untransfected cells were used as a control to generate the background and were subtracted from the test samples. Absorbance was read at a wavelength of 450 nm using a Biotek Synergy HTX plate reader.

### Supernatant ELISA and Fc-binding assays

HeLa cells were transfected with varying concentrations of mRNA, expressing soluble (secreted) gE protein, using Lipofectamine™ MessengerMAX™ according to manufacturer’s instructions. To evaluate expression levels of secreted gE protein, cell supernatants were collected 24 h post transfection and coated on MaxiSorp^TM^ 96-well plates overnight. Alternatively, to assess the ability of secreted gE protein to bind to human Fc, MaxiSorp^TM^ 96-well plates were coated with 4 μg/ml of human IgG1 Fc protein (Abcam), and cell supernatants were added to those pre-coated plates. Soluble gE protein in supernatants or bound to human Fc were then detected by anti-gE monoclonal antibody (GeneTex, 1:1000 dilution), followed by a secondary antibody conjugated to Horseradish Peroxidase (HRP, 1:1000 dilution; Abcam). Tetramethylbenzidine (TMB) was used as a substrate and reaction was stopped with 1 M Phosphoric acid (H_3_PO_4_). Supernatant from un-transfected cells used as a control to generate the background and subtracted from the test samples. Absorbance was read at a wavelength of 450 nm using a Biotek Synergy HTX plate reader.

### LNP formulation

To encapsulate mRNAs in lipid nanoparticles (LNP), purified mRNAs in aqueous solution were mixed with lipid components (the appropriate cationic lipid, a saturated phospholipid, a PEG-lipid and cholesterol) that were present in an organic phase. In this study, two different LNPs were used: one LNP generated using SM102 (1-octylnonyl 8-[(2-hydroxyethyl)[6-oxo-6-(undecyloxy)hexyl]amino]-octanoate; supplied by BroadPharm, San Diego, CA, US) as the cationic lipid (control LNP) and the other LNP generated using a proprietary lipid, developed by NOF Corporation (Tokyo, Japan), as the cationic lipid component. After formation of LNPs, the organic phase was removed by buffer exchange or diafiltration. Thereafter, 1.2 M sucrose was added to a final concentration of 0.1 mg/mL mRNA and 0.3 M sucrose and the mRNA-LNPs were aseptically filtered. These formulated mRNA-LNP particles were then analytically tested for mRNA content, mRNA purity/integrity, LNP size and polydispersity, and thereafter stored at ≤-65°C until final use.

### Mouse studies

All animal work related to this study was conducted following code of ethics for the care and use of animals as guided by the Public Health Service (PHS) Policy on Humane Care and Use of Laboratory Animals, and in compliance with the housing and handling of the animals following the standards of AAALAC (Association for Assessment and Accreditation of Laboratory Animal Care International). Studies were conducted at Charles River Laboratory and hence approved by the institutional animal care and use committee (IACUC) at Charles River Laboratory, ensuring that all experimental procedures were performed in compliance with applicable animal welfare laws and regulations. Animals were housed in suitable facilities with access to food, water, and environmental enrichment. Female animals were used to be consistent with past studies on VZV gE vaccine antigens and characterization of Shingrix^®^ in VZV-primed female mice^19,27,55^. C57BL/6 female mice (Jackson Laboratories), 6-8 weeks of age, were administered with a full human dose (500 µL) of the live attenuated Varicella vaccine (Varivax^®^) by subcutaneous administration, followed by two intramuscular doses of GreenLight’s mRNA-LNP vaccine candidates or Shingrix^®^ in a total volume of 50 µL per mouse (25 µL in each hind limb). All immunizations were performed at 4-weeks intervals. For the Varivax^®^-only and saline control groups, sterile saline was administered, instead of the vaccines, on the immunization days.

To measure acute T cell responses post immunization by intracellular cytokine staining, whole blood was collected at the indicated timepoints by submandibular bleeds or tail nicks. At terminal timepoints, all mice were euthanized via CO_2_ asphyxiation, and blood, spleens and bone marrows were collected for further immunological analysis. All methods are in accordance with ARRIVE (Animal Research: Reporting of In Vivo Experiments) guidelines.

### Recombinant protein production

Recombinant soluble VZV gE protein was generated in-house for mouse serum analysis. The DNA sequence corresponding to the ectodomain of VZV gE (residues 1–538) was codon optimized for expression, synthesized, and cloned into the pcDNA3.1 (+). A thrombin protease cleavage site and a His-tag were incorporated at the C-terminus of the construct to facilitate protein purification. Expi293F cells (Thermo Fisher) were transfected transiently with the plasmids encoding the recombinant gE (rgE) ectodomain using Expi293F transfection kit (Thermo Fisher), and culture supernatant was harvested 3 days after transfection. rgE ectodomain was purified from the clarified supernatant by nickel affinity chromatography with a HisTrap HP 5 mL column using step-elution (Cytiva). The protein was then further purified by Size-exclusion Chromatography (SEC) with a HiPrep 26/60 Sephacryl S-200 column (Cytiva). Based on SEC chromatogram and subsequent SDS-PAGE analysis of the collected fractions, the fractions containing gE were pooled, aliquoted, analyzed for content and purity, and frozen at <-65°C for future use.

### Assessment of gE binding antibodies in sera using ELISA

96-well ELISA plates were coated with recombinant gE protein at 0.5 µg/mL and incubated overnight at 2–8 °C. Next day, plates were washed and blocked for 2 h at room temperature using 1% bovine serum albumin (BSA) in phosphate-buffered saline (PBS). Serial 5-fold dilutions of the serum samples were made, starting at a 1:100 or 1:500 or 1:2500 dilution depending upon the timepoint being measured. After discarding the blocking solution, diluted serum samples were then transferred to blocked plates and incubated for 2 h at room temperature. After incubation, plates were washed with PBS containing 0.05% Tween-20 (PBS-Tween). Goat anti-Mouse IgG (H + L) secondary antibody conjugated with Horseradish peroxidase (HRP) (SouthernBiotech, 1:5000 dilution) was added to the plates for detection and incubated for 1 h at room temperature. Plates were developed with Tetramethylbenzidine (TMB) substrate, and reaction was stopped with 1 M Phosphoric acid (H_3_PO_4_). Absorbance was read at a wavelength of 450 nm using a Biotek Synergy HTX plate reader. Antibody titers were interpolated based on a cut off OD value of 0.2 derived from OD values of blank wells containing no sera.

### Assessment of effector T cell responses by ELISpot Assay

The number of IFN-γ and IL-2 secreting cells were quantified using the Mouse IFN-γ/IL-2 Double-Color ELISPOT kit from ImmunoSpot according to the manufacturer’s instructions. Briefly, 200,000 viable cells from whole blood post RBC lysis were plated onto each well of 96-well, high-protein-binding, PVDF filter plates pre-coated with murine IFN-γ/IL-2 Capture Solution. The cells were stimulated with VZV gE overlapping peptide pool (153 peptides, 15-mers with 11 amino acid overlap) in the presence of anti-CD28/anti-CD49d co-stimulatory antibodies. As a negative control, cells were stimulated with T cell medium only, whereas for the positive control, stimulation was carried out using the polyclonal T cell stimulators PMA (phorbol 12-myristate 13-acetate) + ionomycin along with phytohemagglutinin-L (PHA-L). After overnight (16-18 h) incubation at a 37 °C humidified incubator with 5% CO_2_, plates were developed using the Mouse IFN-γ/IL-2 Double-Color ELISPOT kit from ImmunoSpot according to the manufacturer’s instructions. Plates were scanned and counted utilizing CTL ImmunoSpot® analyzer and ImmunoSpot® 7.0 Pro DC software.

### Evaluation of antibody secreting cells using B cell ELISpot assay

As previously described^23^, PVDF plates were coated with recombinant gE antigen at a concentration of 5 μg/mL overnight at 2–8°C. Next day, 250,000 viable bone marrow cells post RBC lysis were seeded per well onto the coated ELISpot plates and incubated overnight (16-24 h) in a 37 °C humidified incubator with 5% CO_2_. The following day, plates were developed using the MABTech ELISpot Flex: Mouse IgG (ALP) kit according to the manufacturer’s instructions. Plates were scanned and counted utilizing CTL ImmunoSpot® analyzer and ImmunoSpot® 7.0 Pro DC software.

### Assessment of acute and effector memory T cell responses by intracellular cytokine staining

Spleens were harvested from mice as described in the section “Mouse studies”. They were gently pressed through 70 µm strainers using the flat ends of syringe plungers to create single cell suspension. RBCs were lysed, the splenocytes were washed, counted using the Vi-CELL BLU Cell Viability Analyzer and resuspended at a concentration of 5 ×10^6^ viable cells/mL. The cells were next stimulated with VZV gE overlapping peptide pool consisting of 153 peptides (15-mers with 11 amino acid overlap) for 2 h in a 37 °C humidified incubator with 5% CO_2_. As a negative control, cells were stimulated with T cell medium only. For positive controls, splenocytes were stimulated with either polyclonal T cell stimulator PMA+ionomycin alone or PMA+ionomycin along with phytohemagglutinin-L (PHA-L) for IL-2 stimulation. Next, brefeldin A was added and the cells were incubated for another 4 h at 37°C incubator with 5% CO_2_. Splenocytes were incubated with a FcR (Fc Receptor) blocking antibody and stained with the LIVE/DEAD™ Fixable Aqua viability dye (ThermoFisher Scientific) according to manufacturer’s instructions. Cells were washed and stained for surface markers for 30 minutes at 2–8°C in the dark using the following antibodies (Supplementary Table S1): anti-CD3ε, anti-CD4, anti-CD8α, anti-CD62L (for effector memory T cell analysis only), anti-CD44 (for effector memory T cell analysis only). Antibodies against CD19, F4/80, and Gr-1 conjugated to the same fluorochrome were additionally included in the surface marker staining panel to exclude non-T cell populations. Next, cells were washed, fixed and permeabilized using the Foxp3 Staining Buffer Set (Invitrogen) according to manufacturer’s instructions. For Intracellular Cytokine Staining (ICS), surface-labeled splenocytes were labeled with a mixture of anti-IFN-γ, anti-TNF-α, and anti-IL-2 for 30 minutes at room temperature in the dark. Stained samples were finally washed, resuspended in FACS buffer, and data acquired on a BD FACSymphony™ A3 Cell Analyzer. Data was analyzed using FlowJo software (FlowJo, LLC).

### Measure of cytokines in sera using the LEGENDplex™ Mouse Anti-Virus Response Panel

Cytokines were measured from sera collected before immunization and 6 h and 24 h after the first vaccine immunization using the LEGENDplex™ Mouse Anti-Virus Response Panel according to manufacturer’s instructions. Briefly, appropriate standards were prepared according to the kit manual and sera samples were diluted 5-fold before use in the assay. Standards and diluted sera samples were incubated with capture beads for 2 h at room temperature on a plate shaker. Thereafter, beads were spun down, washed and incubated with biotinylated detection antibodies for an hour at room temperature on a plate shaker. Next, PE-labeled streptavidin was added and incubated for 30 minutes at room temperature with shaking. Beads were then spun down, washed twice, and data acquired on a BD FACSymphony™ A3 Cell Analyzer. Data was analyzed using the LEGENDplex™ Data Analysis Software Suite and GraphPad prism version 9.4.0.

### Statistical analysis

Arithmetic or geometric means are represented by symbols or the heights of bars, and error bars represent the corresponding SEM (Standard Error of the Mean). Dotted lines indicate assay’s lower limits of quantification (LLOQ). Two-sided Mann–Whitney U-tests were used to compare two experimental groups and two-sided Wilcoxon signed-rank tests to compare the same animals at different time points. To compare more than two experimental groups, Two-way Analysis of Variance (ANOVA) with Tukey’s multiple comparisons tests were applied. Statistical analyses were done using GraphPad Prism v.9.4.1. *p < 0.05, **p < 0.01, ***p < 0.001, ****p < 0.0001.

### Reporting summary

Further information on research design is available in the Nature Research Reporting Summary linked to this article.

## Supplementary information

SUPPLEMENTARY FILE

REPORTING SUMMARY

## Data Availability

All data generated or analyzed during this study are already included in this manuscript. The raw data is not publicly available because the overall data is proprietary to GreenLight Biosciences Inc. and the NOF LNP information is proprietary to NOF Corporation. GreenLight Biosciences Inc., through the corresponding authors, will make every effort to share additional information or details upon reasonable request.
