# Author Correction: Potent and long-lasting humoral and cellular immunity against varicella zoster virus induced by mRNA-LNP vaccine

**DOI:** 10.1038/s41541-024-01020-w

**Published:** 2024-11-18

**Authors:** Anannya Bhattacharya, Lonzaric Jan, Olga Burlak, Jilong Li, Ghanshyam Upadhyay, Katherine Williams, Jinhui Dong, Harrison Rohrer, Michelle Pynn, Andrew Simon, Nathan Kuhlmann, Sergei Pustylnikov, Mariane B. Melo, Antu K. Dey

**Affiliations:** 1 GreenLight Biosciences Inc., 29 Hartwell Avenue, Lexington, MA 02421 USA; 2 Present Address: Icosavax (AstraZeneca), 1930 Boren Avenue, Seattle, WA 98101 USA

**Keywords:** Viral infection, RNA vaccines

Correction to: *npj Vaccines* 10.1038/s41541-024-00865-5, published online 4 April 2024

In the original version of this Article, Anthony Cataldo was mistakenly included in the Acknowledgements. His name has now been removed from the Acknowledgements and the original Article has been corrected.

