# Supplementary material for: Potent and long-lasting humoral and cellular immunity against varicella zoster virus induced by mRNA-LNP vaccine
Source: NPJ Vaccines. 2024 Apr 4;9:72. doi: 10.1038/s41541-024-00865-5 (PMC10995133; doi:10.1038/s41541-024-00865-5)
Supplement: Supplementary file 1 — SUPPLEMENTARY FILE [file 41541_2024_865_MOESM1_ESM.pdf]

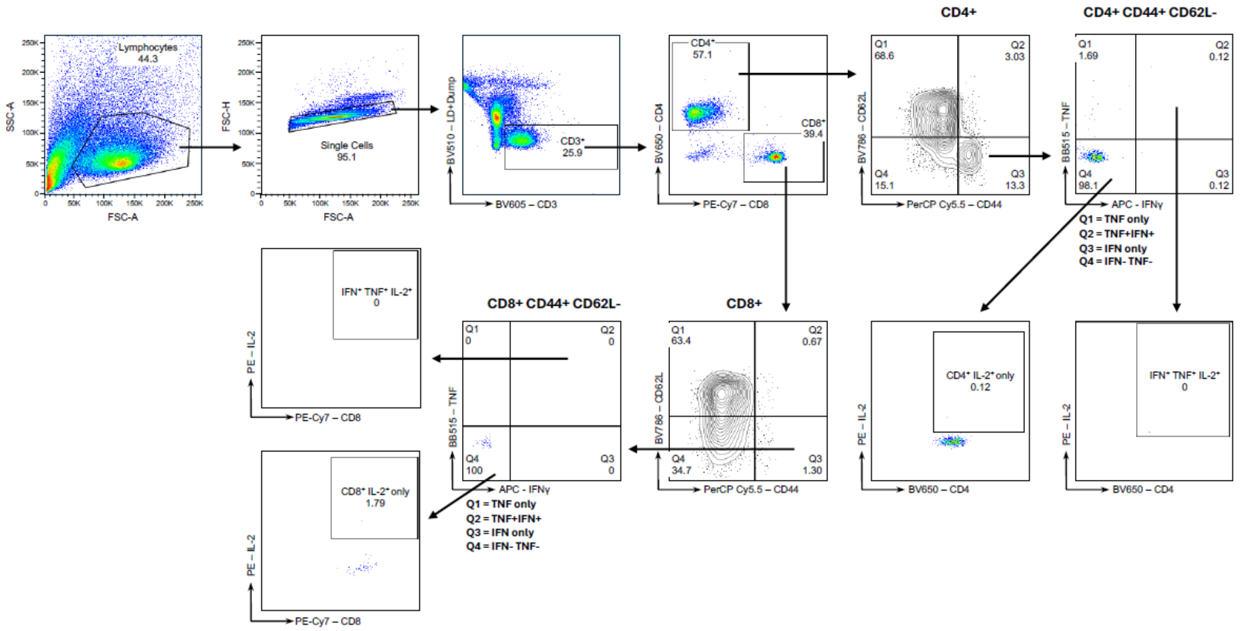

Supplementary Figure 1. Flow cytometry gating strategy to detect antigen specific effector memory T cells. Lymphocytes from peripheral blood cells of immunized mice were gated based on forward scatter (FSC) and side scatter (SSC). After exclusion of doublets, CD3+ T cells were selected and gated for CD4+ or CD8+ subsets, and effector memory cells were identified by presence of CD44 and absence of CD62L surface markers.

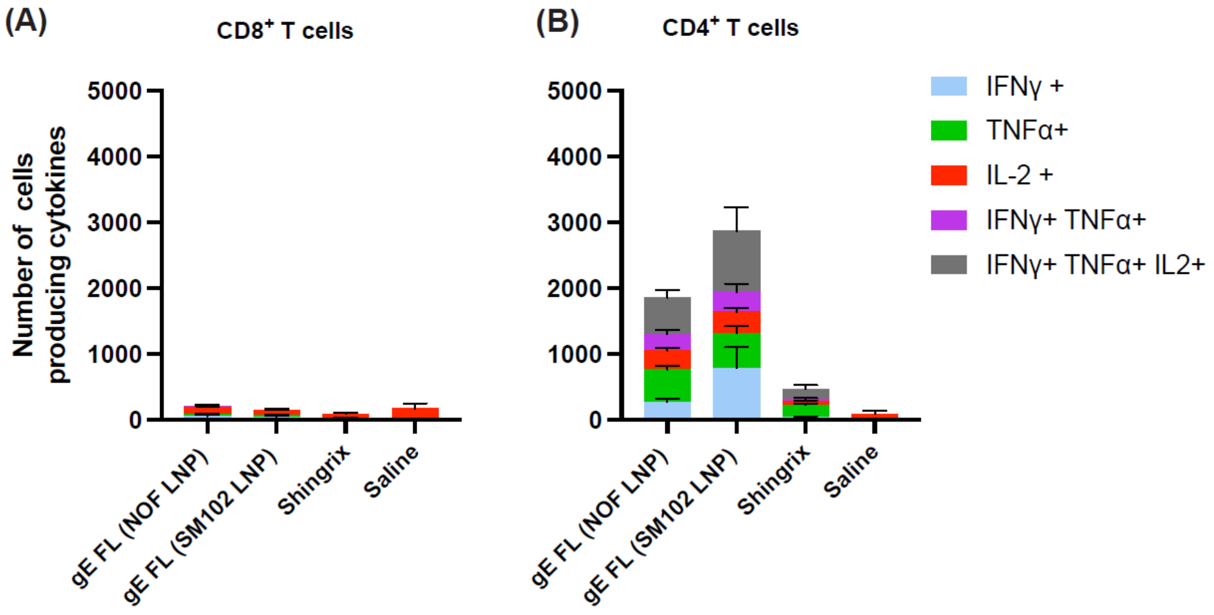

Supplementary Figure 2. CD4^{+} T cell-biased cellular response induced by gE mRNA-LNP vaccines. Animals were divided in groups of 7 and immunized with 2 intramuscular doses, 4 weeks apart, of gE mRNA-LNP (NOF or SM102) or Shingrix®. Mice injected with saline were included as negative controls. One week after the last immunization, spleens were harvested and stimulated with overlapping peptide pools from VZV gE protein, and number of CD8^{+} (A) and CD4^{+} (B) T cells producing IFN-γ, TNF-α and IL-2, was measured by flow cytometry.

Channel	Marker	Dilution	Antibodies (vendor and catalog #)
BV510	CD19	1:200	Biolegend 115546
BV510	F4/80	1:200	Biolegend 123135
BV510	Gr-1	1:200	Biolegend 108438
BV605	CD3 epsilon	1:100	Biolegend 100351
BV785	CD62L	1:100	Biolegend 104440
BV650	CD4	1:200	Biolegend 100546
PerCP-Cy5.5	CD44	1:100	TONBO Biosciences 65-0441-U100
PE-Cy7	CD8a	1:200	TONBO Biosciences 60-0081-U100
FITC	TNF-a	1:50	Biolegend 506304
APC	IFN-g	1:50	TONBO Biosciences 20-7311-U100
PE	IL-2	1:50	TONBO Biosciences 50-7021-U100

Supplementary Table 1. Details of antibodies and their use in flow cytometry.

Attributes tested	Results
mRNA concentration	1.2 mg/mL
Identity (next-generation sequencing)	Alignment % = 99%<br>Reference coverage = 100%<br>Trimmed reads = 280-337k<br>Variants (2-5% MAF) = 0
mRNA purity/integrity (% full length)	84-85%
Poly(A) tail length	105-107 nts
% Capping efficiency	94-98%
Endotoxin (LAL test)	<0.100 EU/mL
RNAse	BLQ*
N1-methyl-pseudouridine (m1Ψ) incorporation	100%
Residual *E. coli* DNA by qPCR	109-185 ng/mL
Residual NTPs	BLQ*

*BLQ = Below limit of quantitation; EU = Endotoxin Units; LAL = Limulus Amebocyte Lysate; NTPs = Nucleotide Triphosphates; nts = nucleotides; MAF = Minor Allele Frequency; qPCR = Quantitative Polymerase Chain Reaction.

Supplementary Table 2: Analytical test results of the three *in vitro* transcribed and purified mRNAs, encoding for gE full length, gE truncated and soluble protein. mRNA concentration was set at 1.2mg/mL. The results that are reported either in ranges or below limit of quantitation are to cover for all three mRNAs.

Attributes tested	NOF-based mRNA-LNPs	SM102-based mRNA-LNPs
Particle size	67-84 nm	84-116 nm
Polydispersity index (PDI)	0.04-0.17	0.04-0.11
mRNA encapsulation (%)	93-98%	91-98%
mRNA purity post encapsulation (%)	80-81%	80-82%
mRNA concentration	0.1mg/mL	0.1mg/mL

Supplementary Table 3: Analytical test results of the six formulated mRNA-LNP vaccine materials. Except for mRNA concentration, which was set at 0.1mg/mL for all materials, all other attribute results are reported in ranges to capture the three gE-antigen mRNAs (encoding for gE full length, gE truncated and soluble protein) formulated in either NOF-LNPs or in SM102-LNPs.
