# Supplementary material for: Potent and long-lasting humoral and cellular immunity against varicella zoster virus induced by mRNA-LNP vaccine
Source: NPJ Vaccines. 2024 Apr 4;9:72. doi: 10.1038/s41541-024-00865-5 (PMC10995133; doi:10.1038/s41541-024-00865-5)
Supplement: Supplementary file 2 — REPORTING SUMMARY [file 41541_2024_865_MOESM2_ESM.pdf]

nature portfolio

Corresponding author(s): Dr Antu (K) Dey

Last updated by author(s): Jan 4, 2024

Reporting Summary

Nature Portfolio wishes to improve the reproducibility of the work that we publish. This form provides structure for consistency and transparency in reporting. For further information on Nature Portfolio policies, see our Editorial Policies and the Editorial Policy Checklist.

Statistics

For all statistical analyses, confirm that the following items are present in the figure legend, table legend, main text, or Methods section.

n/a	Confirmed	
☐	☒	The exact sample size (*n*) for each experimental group/condition, given as a discrete number and unit of measurement
☐	☒	A statement on whether measurements were taken from distinct samples or whether the same sample was measured repeatedly
☐	☒	The statistical test(s) used AND whether they are one- or two-sided<br>*Only common tests should be described solely by name; describe more complex techniques in the Methods section.*
☒	☐	A description of all covariates tested
☐	☒	A description of any assumptions or corrections, such as tests of normality and adjustment for multiple comparisons
☐	☒	A full description of the statistical parameters including central tendency (e.g. means) or other basic estimates (e.g. regression coefficient) AND variation (e.g. standard deviation) or associated estimates of uncertainty (e.g. confidence intervals)
☐	☒	For null hypothesis testing, the test statistic (e.g. *F*, *t*, *r*) with confidence intervals, effect sizes, degrees of freedom and *P* value noted<br>*Give P values as exact values whenever suitable.*
☒	☐	For Bayesian analysis, information on the choice of priors and Markov chain Monte Carlo settings
☒	☐	For hierarchical and complex designs, identification of the appropriate level for tests and full reporting of outcomes
☒	☐	Estimates of effect sizes (e.g. Cohen's *d*, Pearson's *r*), indicating how they were calculated

Our web collection on statistics for biologists contains articles on many of the points above.

Software and code

Policy information about availability of computer code

Data collection	n/a
Data analysis	Data analyzed using Graph Pad Prism 9.4.0 are specifically mentioned in the manuscript

For manuscripts utilizing custom algorithms or software that are central to the research but not yet described in published literature, software must be made available to editors and reviewers. We strongly encourage code deposition in a community repository (e.g. GitHub). See the Nature Portfolio guidelines for submitting code & software for further information.

Data

Policy information about availability of data

All manuscripts must include a data availability statement. This statement should provide the following information, where applicable:

- Accession codes, unique identifiers, or web links for publicly available datasets
- A description of any restrictions on data availability
- For clinical datasets or third party data, please ensure that the statement adheres to our policy

All data generated or analyzed during this study are already included in this manuscript. The raw data is not publicly available because the overall data is proprietary to GreenLight Biosciences Inc. and the NOF LNP information is proprietary to NOF Corporation. GreenLight Biosciences Inc., through the corresponding authors, will make every effort to share additional information or details upon reasonable request.

Research involving human participants, their data, or biological material

Policy information about studies with human participants or human data. See also policy information about sex, gender (identity/presentation), and sexual orientation and race, ethnicity and racism.

Reporting on sex and gender	n/a - no human participants
Reporting on race, ethnicity, or other socially relevant groupings	n/a - no human participants
Population characteristics	n/a - no human participants
Recruitment	n/a - no human participants
Ethics oversight	n/a - no human participants

Note that full information on the approval of the study protocol must also be provided in the manuscript.

Field-specific reporting

Please select the one below that is the best fit for your research. If you are not sure, read the appropriate sections before making your selection.

☒ Life sciences ☐ Behavioural & social sciences ☐ Ecological, evolutionary & environmental sciences

For a reference copy of the document with all sections, see nature.com/documents/nr-reporting-summary-flat.pdf

Life sciences study design

All studies must disclose on these points even when the disclosure is negative.

Sample size	Sample sizes for in vivo studies were selected based on feasibility (handling samples for analysis), previous peer-reviewed publications and specified rationals for our studies
Data exclusions	No data exclusion
Replication	All replicates are described appropriately in the manuscript
Randomization	No randomization
Blinding	No blinding

Reporting for specific materials, systems and methods

We require information from authors about some types of materials, experimental systems and methods used in many studies. Here, indicate whether each material, system or method listed is relevant to your study. If you are not sure if a list item applies to your research, read the appropriate section before selecting a response.

Materials & experimental systems

n/a	Involved in the study	
☐	☒	Antibodies
☐	☒	Eukaryotic cell lines
☒	☐	Palaeontology and archaeology
☐	☒	Animals and other organisms
☒	☐	Clinical data
☒	☐	Dual use research of concern
☒	☐	Plants

Methods

n/a	Involved in the study	
☒	☐	ChIP-seq
☐	☒	Flow cytometry
☒	☐	MRI-based neuroimaging

Antibodies

Antibodies used	Antibodies for Western blot:<br>anti-gE primary antibody (Varicella Zoster virus gE antibody; Catalog No. GTX64187; GeneTex) and a goat anti-mouse fluorescently labeled secondary antibody (Catalog No. SA5-10264; Invitrogen).

Antibodies for ELISAs: Antibodies for Western:
anti-gE primary antibody (Varicella Zoster virus gE antibody; Cat. No. GTX64187; GeneTex), anti-mouse antibody conjugated to HRP (Catalog No. ab6728; Abcam), Goat anti-Mouse IgG (H+L) conjugated to HRP (Catalog No. 1036-05, SouthernBiotech)

Antibodies for Flow Cytometry (vendor, catalog #):
Biolegend 115546, Biolegend 123135, Biolegend 108438, Biolegend 100351, Biolegend 104440, Biolegend 100546, TONBO Biosciences 65-0441-U100, TONBO Biosciences 60-0081-U100, Biolegend 506304, TONBO Biosciences 20-7311-U100, TONBO Biosciences 50-7021-U100. Please see Supplementary Table S1 for additional details.

Validation

All antibodies were purchased from external vendors with product sheet to validate specificity and use.

Eukaryotic cell lines

Policy information about cell lines and Sex and Gender in Research

Cell line source(s)

HEK293FT cell lines (Catalog number: R70007) was sourced from ThermoFisher.
HeLa cell lines (ATCC number: CCL-2) was sourced from ATCC.

Authentication

Vendor documentation for both cells were accepted for our acceptance of authenticity; growth and morphology of the cell lines in our laboratory also supported the appropriateness of the cell lines

Mycoplasma contamination

Both cell lines, HEK293FT and HeLa, were expanded and tested for Mycoplasma . Both cell lines were Mycoplasma negative.

Commonly misidentified lines
(See ICLAC register)

none

Animals and other research organisms

Policy information about studies involving animals; ARRIVE guidelines recommended for reporting animal research, and Sex and Gender in Research

Laboratory animals

C57BL/6 female mice (Jackson Laboratories), 6-8 weeks of age

Wild animals

n/a

Reporting on sex

Female

Field-collected samples

Study did not involve samples collected from the field; n/a

Ethics oversight

All animal work related to this study was conducted following code of ethics for the care and use of animals as guided by the Public Health Service (PHS) Policy on Humane Care and Use of Laboratory Animals, and in compliance with the housing and handling of the animals following the standards of AAALAC (Association for Assessment and Accreditation of Laboratory Animal Care International). Studies were conducted at Charles River Laboratory and hence approved by the institutional animal care and use committee (IACUC) at Charles River Laboratory, ensuring that all experimental procedures were performed in compliance with applicable animal welfare laws and regulations. Animals were housed in suitable facilities with access to food, water, and environmental enrichment.

Note that full information on the approval of the study protocol must also be provided in the manuscript.

Plants

Seed stocks

n/a

Novel plant genotypes

n/a

Authentication

n/a

Flow Cytometry

Plots

Confirm that:

☒ The axis labels state the marker and fluorochrome used (e.g. CD4-FITC).

☒ The axis scales are clearly visible. Include numbers along axes only for bottom left plot of group (a 'group' is an analysis of identical markers).

☐ All plots are contour plots with outliers or pseudocolor plots.

☒ A numerical value for number of cells or percentage (with statistics) is provided.

Methodology

Sample preparation	Sample preparation details are provided in the METHODS section of our manuscript.
Instrument	BD FACSymphony™ A3 Cell Analyzer
Software	FlowJo software (FlowJo, LLC)
Cell population abundance	We analyzed 1,000,000 events from each sample. Cell viability was above 95% in all samples analyzed. Samples had in average 20% of CD3+ T cells that were subsequently analyzed for expression of activation and memory markers. No cell sorting was performed.
Gating strategy	Supplementary Figure S1 for our Flow cytometry gating strategy (to detect antigen specific effector memory T cells)

☒ Tick this box to confirm that a figure exemplifying the gating strategy is provided in the Supplementary Information.
